# Elevational Patterns of Plant Richness in the Taibai Mountain, China

**DOI:** 10.1155/2014/309053

**Published:** 2014-10-28

**Authors:** Lili Tang, Tanbao Li, Dengwu Li, Xiaxia Meng

**Affiliations:** ^1^College of Forestry, Northwest A & F University, Yangling 712100, China; ^2^Northwest Institute of Forest Inventory, Planning and Design, SFA, Xian 710078, China

## Abstract

The elevational distribution of plant diversity is a popular issue in ecology and biogeography, and several studies have examined the determinants behind plant diversity patterns. In this study, using published data of the local flora of Taibai Mountain, we explored the effects of spatial and climatic factors on plant species richness. We also evaluated Rapoport's elevational rule by examining the relationship between elevational range size and midpoint. Species richness patterns were regressed against area, middle domain effect (MDE), mean annual temperature (MAT), and mean annual precipitation (MAP). The results showed that richness of overall plants, seed plants, bryophytes, and ferns all showed hump-shaped patterns along the elevational gradient, although the absolute elevation of richness peaks differed in different plant groups. Species richness of each plant group was all associated strongly with MAT and MAP. In addition to climatic factors, overall plants and seed plants were more related to area in linear regression models, while MDE was a powerful explanatory variable for bryophytes. Rapoport's elevational rule on species richness was not supported. Our study suggests that a combined interaction of spatial and climatic factors influences the elevational patterns of plant species richness on Taibai Mountain, China.

## 1. Introduction

The spatial patterns of species richness and its underlying mechanisms have been one of the hotspots in ecology [1–3]. In the past decades, many studies on species richness patterns were carried out along latitudinal and depth gradient [4–10]. However, as a surrogate of latitude, patterns of species richness and their ecological determinants in mountain regions were paid more attention by scholars in the recent ten years [11–14]. Generally speaking, the ecological environment of mountain regions has strong environmental heterogeneity due to the complexity of physical conditions; thus the flora and fauna are very rich in mountains. Furthermore, Lundholm [15] studied forty-one observational and eleven experimental reports that quantified plant species diversity and heterogeneity of spatial environment and found that positive heterogeneity-diversity relationships were very common, confirming the importance of niche differentiation in species diversity patterns. Elevation is one of the decisive factors for species richness in mountain ecosystems, presenting drastic climate changes (temperature, water) as well as overall area [13, 16, 17]. Besides, Moeslund et al. [18] provided an overview of the evidence for the different mechanisms involved in topography's control of local patterns in potential vegetation drivers and found that topography is an important factor for local plant diversity patterns across most habitats, even in relatively flat lowland areas. In the last few years, many researches demonstrated the elevational patterns of mammals [19–24], birds [25–27], insects [28–30], and plants [31–35] in different taxa and regions.

It is well known that elevational pattern of species diversity is similar to latitudinal pattern;, that is, with the elevation increasing and heat decreasing, species richness decreases [36–38]. However, some studies suggest that the highest species richness appears at midelevational regions [22, 29]. What is more, the hump-shaped pattern was found to be most common in research reports, accounting for almost half of the observed studies [12, 39]. Stevens proposed that species richness was affected by interaction among temperature, precipitation, competition, and the historical processes, yet there was no specific relationship between elevation and species richness [11].

Many hypotheses, which have been proposed to explain the species richness patterns, are divided into two broad categories, namely, Rapoport's elevational rule and those considering spatial and climatic factors. It has been widely accepted by biogeographers and ecologists that the area of elevational band was a significant factor for species distribution pattern and it could explain a large proportion of the variation in species richness [1, 25, 40–43]. Previous researches also indicated that the available area of different elevations varied greatly in mountainous regions [13, 29, 35, 44]. Biogeographically, larger areas are often considered to have more species because they have a higher carrying capacity for species [1, 45]. Another spatial factor is the middle domain effect. Due to geometric constraints or hard boundaries on species ranges within a bounded domain, overlap degree of distribution in different species is smaller in the edge region, but larger in the center region, yielding a middomain peak in species richness [46–48]. Several studies suggested that MDE was a powerful explanatory variable for the elevational patterns of species richness [22, 31, 49].

Climatic variables are critical in species elevational patterns of various living organisms [50]. The distribution range margins of individual species were controlled directly or indirectly by climatic factors when they exceed the physiological tolerances of species [24, 40]. Recently, some studies have found that species richness along elevational gradients commonly correlated with climatic factors like temperature and precipitation [31, 51]. What is more, many scholars suggested that the hump-shaped pattern of species appeared due to the unimodal distribution of precipitation along the elevational gradient [12, 20, 52].

Rapoport's elevational rule suggests that species in high elevations (they could tolerate extreme climatic conditions) have a broader distribution range than species in lowland. Consequently, species richness is inflated at low elevations and then decreases with increasing elevation [11]. Although large numbers of researches have been conducted to test Rapoport's elevational rule, its conclusions and evidences are rather controversial; some results supported Rapoport's elevational rule [29, 53–55], while some did not follow this rule [25, 56–58].

In this study, we examined the pattern of plant species richness along the elevational gradient of Taibai Mountain. Qin et al. [59] only studied the effects of area and MDE on distribution pattern of plants in Taibai Mountain without considering the climatic factors which obviously control species distribution and richness in many areas. Based on the power law of species-area relationship (SAR), the metabolic theory of ecology (MTE), and the middle domain effect hypothesis (MDE), Chi and Tang [60] tended to explore the determinant mechanisms of plant species distribution pattern of Taibai Mountain, but they failed to test Rapoport's elevational rule which is closely related to the elevational pattern of species richness [11]. Thus, using the well-documented local flora information, the main aims of our study are (1) to describe the elevational patterns of species richness for overall plants, seed plants, bryophytes, and ferns of Taibai Mountain, (2) to evaluate the influence of spatial effects (area and MDE) and climatic factors (MAT—mean annual temperature and MAP—mean annual precipitation) on elevational patterns of plant species richness, (3) to statistically evaluate the respective contributions of those spatial and climatic factors, and finally (4) to test Rapoport's elevational rule by examining the relationship between midpoint and elevational range sizes of plant species.

## 2. Materials and Methods

### 2.1. Study Area

**Figure 1:**
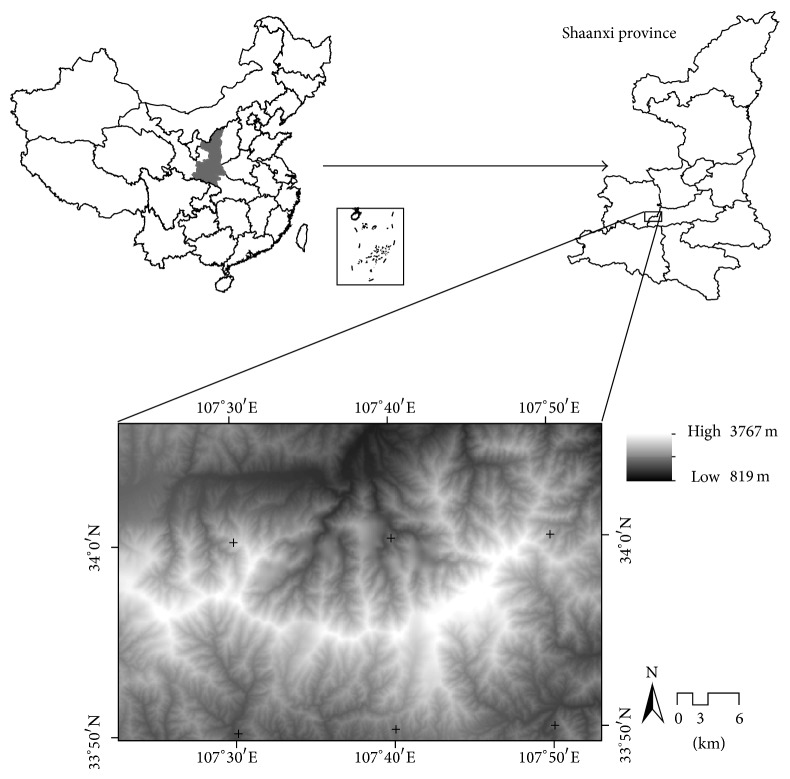
Geographic location and topography of Taibai Mountain.

Taibai Mountain (107°22′–107°51′ E, 33°49′–34°05′ N), the middle of the Qinling Mountains in Shaanxi province of China, covers Taibai County, southern part of Mei County and southwestern part of Zhouzhi County; the elevational gradient of study region extends from 819 to 3767 m a.s.l. (Figure 1). The Nature Reserve of Taibai Mountain was designated in September 1965 by the government of Shaanxi province. The flora of Taibai Mountain is extremely rich, including 1783 seed plant species (597 genera and 126 families), 325 bryophyte species (142 genera and 62 families), and 110 fern species (40 genera and 21 families) [61]. It is also known as an international significant area of biological diversity in China.

The Taibai Mountain belongs to a mountain ecoregion and comprises warm temperate zone, temperate zone, cool temperate zone, and subalpine zone [62]. The vegetation can be divided into four major zones along an elevational gradient. These elevational vegetation zones include (1) deciduous oak forest (800–2300 m a.s.l.) dominated by* Quercus wutaishanica* Mayr.,* Quercus aliena* Bl. var.* acuteserrata* Maxim.,* Quercus variabilis* Blume., and* Pinus armandii* France; (2) birch forest (2300–2800 m a.s.l.) characterized by* Betula utilis* D. Don,* Betula albosinensis* Burk., and* Pinus armandii* France; (3) coniferous forest (2800–3400 m a.s.l.) dominated by* Larix chinensis* Beissn and* Abies fargesii* Franch; (4) subalpine meadow (3400–3767 m a.s.l.) dominated by* Rhododendron capitatum* Maxim. and* Salix cupularis* Rehd. [61].

### 2.2. Plant Data

A database generated from “*Biodiversity, Conservation and Management of Taibaishan Nature Reserve*” [61] and “*Flora of Qinling*” [63], which is based on substantial field surveys, was used for analyses in this study. The information in this database includes species identity, genus and family of each species, and their elevational distribution range and plant groups (overall plants, seed plants, bryophytes, and ferns). The database does not include the data of population sizes of species and thus we cannot analyze the effect of sampling individuals on species richness.

In order to analyze the elevational changes in species richness, the study region was divided into 30 elevational bands of intervals from 800 m a.s.l. to 3767 m a.s.l. The species richness was defined as the number of species in every 100 m interval. We interpolated the presence of each species using the recorded elevational range (between maximum and minimum elevations). This method assumes that species are continuously distributed between their lower and upper limits, and interpolation has been commonly used in many recent studies of elevational patterns of species richness [35, 64, 65]. To improve the quality of data analyses, we referred to other similar researches [11, 58, 66–68] and handled data as follows: (1) we got rid of the species without clear record of elevational distribution range (2) for species with only one elevational record; we used this record elevation as the midpoint and the elevational range size was broadened to 100 m. (3) for species with clear upper and lower elevation limited; we got 100 m as the height unit using rounding method. In this study, our database recorded detailed elevational distribution limits for 1858 kinds of plant species (containing 1491 seed plant species, 257 bryophyte species, and 110 fern species).

### 2.3. Spatial Effect

**Figure 2:**
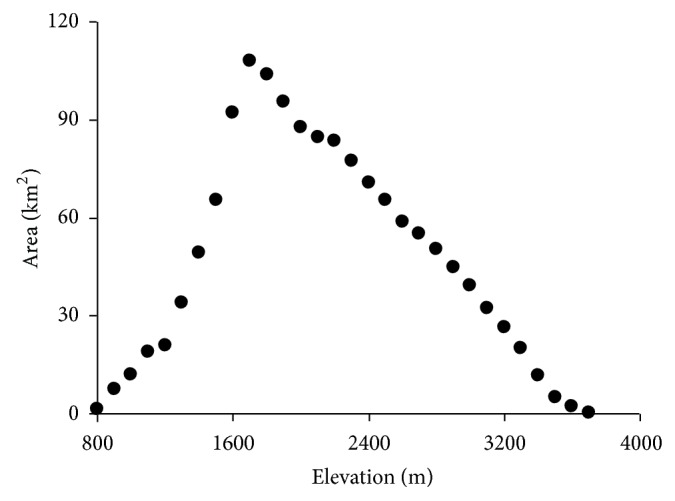
Variation of area along the elevational gradient in the Taibai Mountain.

Area is one of the most important factors determining species richness patterns [1]. To test the relationship between area and species richness, the area of each elevational band was calculated by geographic information system software (Figure 2). The DEM data was provided by the International Scientific & Technical Data Mirror Site, Computer Network Information Center, Chinese Academy of Sciences (http://modis.datamirror.csdb.cn/). The resolution of this DEM data was 90 m ∗ 90 m and the area is a product of grid number by grid area. As area and species richness do not have a linear relationship, we used a log-transformed area as the explanatory variable [49, 69].

In recent years, a geometrical null model (MDE) in which ranges of observed species are randomly placed has commonly been used to generate the pattern of predicted species richness [47, 48]. While there is a heated debate on the significance and implications of this model [70–72], several studies indicated that MDE in some cases explained almost all variation in elevational species richness patterns, especially combined with the influence of area [29, 35, 40]. An MDE null model was used to test the influence of geometric constraints on the species richness along the elevational gradient [70, 73, 74]. We used RangeModel software 5 [73] to generate the null distributions. The simulation process was repeated 5000 times in computer; the predicted mean richness and its 95% confidence intervals were used to assess the effects of geometric constraints on the spatial patterns of species richness.

### 2.4. Climatic Factors

**Figure 3:**
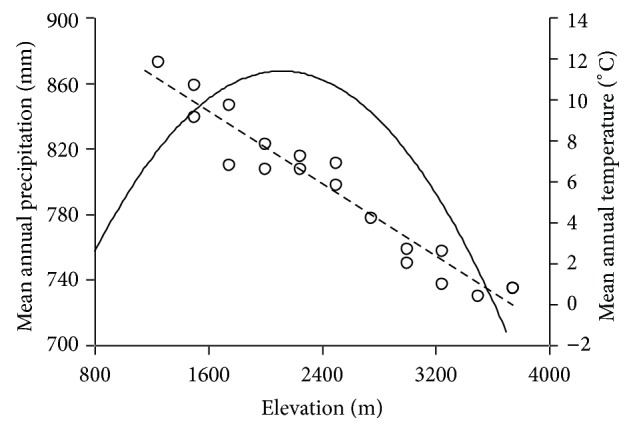
Relationships between elevation and mean annual temperature (MAT) (dash line) and mean annual precipitation (MAP) (solid line).

Mean annual temperature (MAT) and precipitation (MAP) were two major climatic variables as potential explanations for variations of plant species richness [31]. Due to complex terrain conditions and rare meteorological stations, it is difficult to obtain the microclimate data at different elevations in mountain regions. In order to obtain the accurate temperature information of different elevational band, the MAT data used in this study came from the field measurement of Ren et al. from 2001 July to 2002 July [61]. In the north and south slopes of Taibai Mountain, 18 miniature automatic meteorological recorders were placed at every 250 m along an elevational gradient. Then the fitting equation between elevation and MAT was established; MAT of each elevational band can be calculated based on this equation (Figure 3). The MAP data were derived from yearbooks among Zhouzhi County, Taibai County, and Mei County. According to the relationship between MAP and elevation in mountain regions on the following equation [75], we calculated MAP of each elevational band below 2000 m a.s.l. in north slope and 2300 m a.s.l. in south slope, respectively:

(1) $$\begin{array}{c} P_{Z}=P_{h_{0}}+a\left[\left(2H-Z\right)\times Z-\left(2H-h_{0}\right)\times h_{0}\right], \end{array}$$

where *P*_*Z*_ is a certain elevation (*Z*) precipitation and *P*_*h*_0__ is a reference elevation (*h*_0_) precipitation below the maximum precipitation of height (*H*); *a* is a parameter related to regional characteristics (in south slope *a* = 7.778 × 10^−5^, in north slope *a* = 4.938 × 10^−5^). Above 2000 m a.s.l. in north slope and 2300 m a.s.l. in south slope, the MAP of each elevational band was predicted by extending the fitting line. The mean precipitation between north and south slopes of Mountain Taibai was calculated and used to analyze the impact of precipitation on species richness along the elevational gradient (Figure 3).

### 2.5. Statistical Methods

In this study, the relationship between the explanatory variables and species richness was calculated for each individual variable using simple linear regression then overall using stepwise multiple linear regression analyses. The linear models were examined both with and without the influences of MDE as an explanatory variable. However, there may be a problem with using linear models to assess the importance of variables in explaining elevational richness patterns, especially if the patterns are hump-shaped. Biologically, it is entirely feasible that species richness is limited at low elevations by a different factor (e.g., drought) than at high elevations (e.g., low temperatures) [65]. What is more, a linear model cannot better describe the relationship between the species richness and temperature [49]. Thus, we also used a polynomial model (including a quadratic term regression function [in its general form: *f* = *b*_0_ + *b*_1_∗variable + *b*_2_∗variable^2^]) to assess the effect of explanatory variable on species richness [49]. We considered *F*-significance ≤0.05 as significant as standard in all analyses [76] and model fits were assessed using the determinate coefficient. All statistical analyses were performed in SPSS 17.0 software.

### 2.6. Rapoport's Elevational Rule

Since Rapoport's rule was proposed by Stevens, four methods have been used to verify Rapoport's rule successively [77–80]. In this study, the relationship between midpoint and elevational range sizes of plant species was examined by “midpoint method,” which can overcome the statistical nonindependence for spatial data [78, 81]. While Rapport's rule predicts that elevational amplitudes should increase with elevation [11], it has also been predicted that range amplitudes are the highest in the middle of gradients because wide-ranging species must occur in intermediate region due to geometrical constraints [46]. Thus we propose two alternative hypotheses and test them by contrasting linear and polynomial models. Both simple linear and second-order polynomial regression analyses were used to evaluate the relationship between elevational range size and midpoint. If the correlation between these two variables was positive in simple linear regression analysis, Rapoport's elevational rule would be supported.

## 3. Results

### 3.1. Species Richness Patterns along the Elevational Gradient

The total number of plant species used in the analyses was 1858. More than half of these species were seed plants (80.3%, 116 families, 524 genera, and 1491 species), while the number of bryophytes (48 families, 117 genera, and 257 species) and ferns (21 families, 40 genera, and 110 species) accounts for 13.8% and 5.9%, respectively (Table 1).

**Figure 4:**
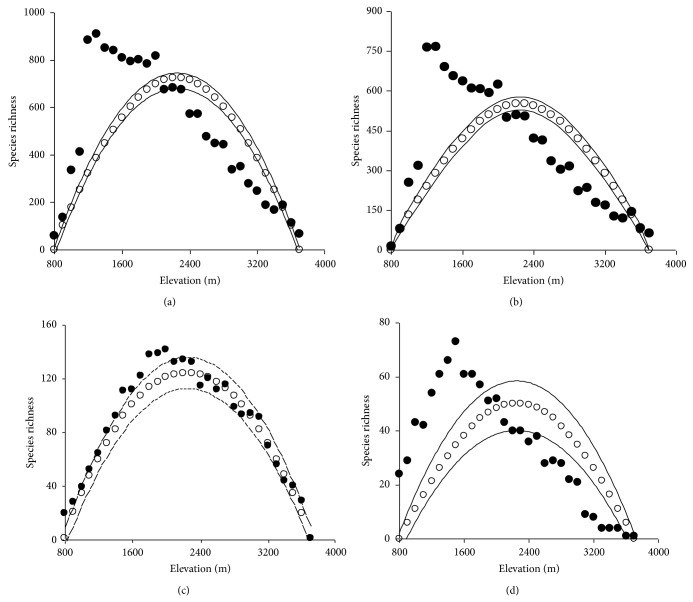
Elevational patterns of observed species richness and predicted richness (computed from 5,000 randomizations) in Taibai Mountain. Dot represents observed richness, solid line represents predicted richness by RangeModel based on middomain effect, and dashed lines represent the interval of predicted richness with 95% confidential. Overall plants (a), seed plants (b), bryophyte (c), and fern (d).

Species richness of overall plants exhibited a clear hump-shaped pattern peaking at 1200–1300 m a.s.l. along the elevational gradient in Taibai Mountain. The elevational patterns of other plant groups were similar with the distribution of overall plants, although the elevation of richness peak varied somewhat; namely, richness of seed plants peaked at 1200–1300 m a.s.l., lower than the peak elevation of bryophytes and ferns (between 1800 m and 2000 m a.s.l. for bryophytes and 1500 m a.s.l. for fern plants) (Figure 4). In these hump-shaped patterns, the distribution trend of bryophytes was mild, whereas the other three groups increased steeply at low elevation and then slowly declined at high elevation after reaching the peak value, especially for overall and seed plants (Figure 4). Furthermore, seed plants, bryophytes, and ferns had different contributions to the richness pattern of overall plants; the correlation coefficient between seed plants and overall plants was the highest (*R* ^²^ = 0.99, *P* < 0.001), followed by ferns (*R* ^2^ = 0.81, *P* < 0.001) and bryophytes being the lowest (*R* ^²^ = 0.57, *P* < 0.001).

By using the MDE null model for simulation, the results showed that the patterns of predicted richness were symmetrically peaking at 2200~2300 m a.s.l. along the elevational gradient, while the observed richness of plant groups (except for bryophytes) had partial peak patterns and the maximum value appeared at the first third or quarter of the elevational transect (Figures 4(a), 4(b), and 4(d)). Moreover, the analysis indicated that more than 40% of the data points fell outside the 95% confidence interval of the MDE null model for species richness (93%, 87%, 40%, and 90% for overall plants, seed plants, bryophytes, and ferns, resp.). Even so, there still is a significant correlation between predicted and observed plant species richness in simple linear regression (Table 2).

### 3.2. Patterns of Richness with Spatial and Climatic Factors

With increasing elevation, the area of each elevational band increased sharply and then decreased after reaching the peak at 1700 m a.s.l., showing a hump-shaped pattern (Figure 2). By extending the fitting line of the equation between MAP and elevation, MAP also depicted a hump-shaped curve along the elevational gradient with a peak (about 900 mm*·*year^−1^) at 2100~2200 m a.s.l. The MAT of Taibai Mountain monotonically decreased with increasing elevation and the decline rate was 0.44°C*·*100 m^−1^ (Figure 3).

Based on simple linear regressions, the results showed that almost all of the variables considered were significantly correlated with species richness (Table 2). Specifically, area and MAP had a relative higher coefficient of determination with overall plants and seed plants, and bryophytes highly correlated with area, MDE, and MAP, whereas ferns were more related to MAT and MAP. For overall plants and seed plants, MDE and MAP were included in the stepwise multiple regressions (model A) and together explained 72% and 65% of the variation in species richness of these two plant groups, respectively. As for bryophytes, MDE and MAT were included in model A, explaining 96% of the variation in specie richness, while MAT and MAP were included in model A and explained 82% of the variation in fern species richness. However, when excluding the effect of MDE, all final models (model B) only included MAT and MAP (Table 2). Moreover, the final model fits (*R* ^2^) among different plant groups were similar or slightly lower than those of model A (71%, 63%, 96%, and 82% for overall plants, seed plants, bryophytes, and ferns in model B, resp.).

In the second-order polynomial regressions, the correlation coefficients of predictor variables expected for MAT did not change much compared to those in sample linear regressions (Table 3). However, MAT, which was poorly correlated with species richness in the sample linear regressions (Table 2), was highly correlated with species richness and was a robust factor in curvilinear regressions (Table 3). The regression value of MAT increased from 0.24 (*P* < 0.01), 0.26 (*P* < 0.01), 0.01 (not significant), and 0.60 (*P* < 0.001) in a linear model to 0.71 (*P* < 0.001), 0.63 (*P* < 0.001), 0.96 (*P* < 0.001), and 0.82 (*P* < 0.001) in a second-order polynomial model for overall plants, seed plants, bryophytes, and ferns, respectively.

### 3.3. Rapoport's Elevational Rule

**Figure 5:**
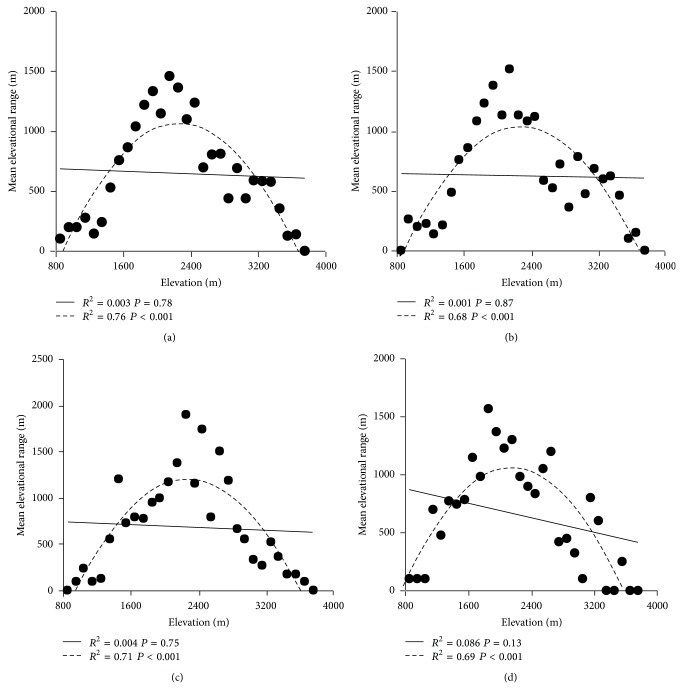
Midpoint method to test Rapoport's elevational rule. The straight and the dotted curved lines show the simple linear and the second-order polynomial regression models, respectively. Overall plants (a), seed plants (b), bryophyte (c), and fern (d).

By using midpoint method [78], the results depicted a hump-shaped relationship between elevational distribution range and midpoint; namely, elevational range increased at low elevation and then declined at the high elevation after peaking at intermediate elevations (Figure 5). The two-order polynomial regression showed that elevational range size was significantly correlated with elevation (*P* < 0.001); furthermore the fitting curve was well consonant with the data points (*R* ^2^ = 0.76, 0.68,0.71,0.55 for overall plants, seed plants, bryophytes, and ferns, resp.). On the contrary, there was a negative and insignificant (*P* > 0.01) correlation between these two variables in simple linear regression model and the correlation coefficient was very low (*R* ^2^ = 0.003, 0.001,0.004,0.081 for overall plants, seed plants, bryophytes, and ferns, resp.) (Figure 5). From both linear regression and nonlinear regression, the results did not support the first hypotheses that elevational amplitude should increase with elevation [11].

## 4. Discussion

The elevational patterns of species richness and their potential causes have been a popular issue in ecology and biogeography. Some studies indicate that species richness in mountain area monotonically decreases with increasing elevation [11, 82], while some suggest that species richness peaks at intermediate elevational region [22, 29, 47]. In our study, plant species richness in Taibai Mountain exhibited a clear hump-shaped pattern along the elevational gradient (Figure 4). Such a pattern was also identified for three plant groups (seed plants, bryophytes, and ferns), although the absolute elevation of richness peaks differed among the plant groups. At the most general level, our study adds the evidence that on high mountain plant species richness peaks at midelevations and shows a hump-shaped pattern.

In this study, a species was considered as present at every 100 m between its lower and upper limits of recorded elevational ranges. Such interpolation method has been widely used to study species distribution patterns along elevational gradient [25, 29, 64]. Some studies suggest that interpolation may artificially increase richness to a higher degree at midelevation area, because species are only strictly observed at the extreme ends of elevational ranges [64]. However, we have no evidence that species are not found within the interpolated range, which may not significantly alter the trend of species distribution [83]. Furthermore, Wang et al. [35] found that species with range size of ≤100 m and ≤200 m without interpolation both showed hump-shaped patterns in Gaoligong Mountains, implying that the effect of interpolation method on the hump-shaped patterns may not be substantial.

Except for interpolation method, some previous studies have also reported that the elevational patterns of plant species are affected by a series of factors, containing spatial, climatic, historical factors, and human impacts [39, 49, 51, 64, 84, 85]. Below, we discuss the influence of area, MDE, MAT, MAP, and Rapoport's elevational rule on plant species richness of Taibai Mountain in detail.

### 4.1. Area

Area is a critical parameter to determine elevational species richness patterns [12, 86], and it can be explained by several mechanisms [1]. The species-area relationship is the most common theory accepted by scholars, although the exact structure relationship has been under discussion [86–88]. Some studies have suggested that species richness patterns in mountain area were, at least partially, affected by area [13, 25, 44, 89]. In our study, the results indicated that the importance of area effect was different in different plant groups. Compared with other factors, area was a relative weak factor for bryophytes and ferns species, while species of overall plants and seed plants were more related to area although it was excluded in the stepwise multiple regression analysis. Some studies also found that area had a significant impact on seed plants pattern [35, 60]. Recently, Lee et al. [31] have supposed that area effect may be masked by the MDE, in their study of plant species richness along the ridge of the Baekdudaegan Mountains, South Korea. However, our results did not confirm this supposition because when we excluded the MDE effect in the multiple regression model (model B in Table 2), the area variable was also excluded from the analysis. Instead, due to the high correlation between area and MAP (*R* ^2^ = 0.87, *P* < 0.001), we suspect that area effect may be substituted by the strength of MAP in the stepwise multiple regression models, at least for the overall plant and seed plant species in this study.

### 4.2. MDE

The geometric constraint may be another spatial factor causing the variation of species richness patterns, and many studies have confirmed that MDE has a strong explanatory power on hump-shaped patterns [31, 35, 49, 84]. In this study, MDE was a powerful factor on pattern of bryophytes and the null model fitted the observed species of bryophytes well (Figure 4(c)), whereas it was a weak predictor of ferns richness. For overall and seed plants, although MDE had a significant influence (*P* < 0.05) on species richness both in the simple linear and in stepwise multiple regressions, the correlation coefficient between MDE and species was low (*R* ^2^ < 0.5), indicating that the MDE may be a relative weak factor to the distribution patterns (Table 2). Analyzing the published papers on biodiversity patterns of small mammals along elevation gradient, McCain [23] found that the average *R* ^2^ of the regression models between observed species richness and MDE was very small (0.295 for gamma diversity). In this study, the *R* ^2^ of the simple linear regression model between MDE and species richness (except for bryophytes) was also low (*R* ^2^ = 0.45, 0.36,0.22 for overall plants, seed plants, and ferns).

We found a big deviation between observed and predicted richness, and more than 40% of the data points fell outside the 95% confidence interval (Figure 4). Similar deviation between observed and predicted richness was also found in other studies [31, 49, 90]. Some scholars suggest that the large deviation may be due to a large proportion of such species that present in only one or two samples [31, 91], while some propose that it may be caused primarily by an accumulation of species with narrow elevational amplitudes at either end of the gradient [49]. Moreover, the degree of deviation may also imply that other elements such as ecological, historical and evolutionary factors could explain the species distribution pattern [85]. In our study, we suggest that the great degree of deviation at the front half of transects may reflect the area effect on species richness patterns, at least for overall plants and seed plants.

Recently, many researches have confirmed the prediction of the MDE hypothesis that MDE is stronger for the diversity patterns of species with large elevational ranges than species with narrow ranges [31, 35, 48, 49, 84]. In this study, the explanatory power of MDE on pattern of bryophytes was stronger than the pattern of seed plants (Table 2). One reason may be that the proportion (77%) of the number of bryophytes whose elevational amplitudes was less than half of the whole transect was significantly smaller than that (90%) of seed plants [60].

### 4.3. Climate

Climate is another factor considered in this study and many studies have suggested that distribution patterns of species richness could be well explained by climatic variables such as PET (potential evapotranspiration), MAT, MAP, or humidity [31, 49, 51, 92]. By comparing Taiwan species distribution with other same latitude mountains, Zhang et al. [93] found that the formation of elevational richness patterns of plants may be closely associated with the elevational patterns of precipitation. The peak of the MAP in Taibai Mountain occurred at intermediate elevational area (around 2100~2200 m a.s.l.) (Figure 3), easily to form cloud zone so that large quantity of water is deposited directly onto vegetation from clouds and light mist, promoting plant growth and development. Moreover, the statistic results indicated that MAP was a strong predictor for species richness of overall plants, seed plants, and ferns both in simple linear regression and multiple regression models (Table 2). For bryophytes, MAP was highly correlated with species richness in simple linear regression whereas it was excluded in multiple regression model (model A). However, MAP had a significant correlation with bryophyte richness in model B without the effect of MDE. This apparent contradiction may be due to the high correlation between MAP and MDE (*R* ^2^ = 0.89, *P* < 0.001 for bryophytes); thus we suspect that the influence of MAP was masked by the strength of the MDE in multiple regressions, at least for bryophyte species in this study.

By studying the elevational species richness patterns of different plant groups in Nepal, Central Himalaya, Grau et al. [94] found an interesting order that the maximum species richness occurred at decreasing altitudes, starting with bryophytes at highest elevations, followed by ferns and finally vascular plants towards lower altitudes. We also get the same result in this study. The maximum richness of bryophytes is observed between 1800 m and 2000 m a.s.l., higher than the peak elevation of fern plants (1500 m a.s.l.) and seed plants (1200–1300 m a.s.l.) (Figure 4). The order of this appearance may be related to the environmental humidity. Compared with vascular plants (including seed plants and ferns), bryophytes are smaller in morphology and have no real roots to store water and without conducting tissues to transport water, consequently, more highly depend on soil-water availability and air humidity. On the other hand, compared with pteridophytes, seed plants are better able to adapt to arid conditions by the special morphology and life-history strategies, such as annual life style and pronounced succulence, deep-rooted perennials [95]. Additionally, Grau et al. [94] interpreted these differences from the perspective of driving forces of terrestrial plant evolution and suggest that the order of appearance from higher to lower elevation might reflect the sequence of plant evolution on the basis of the phylogeny proposed by Oliver et al. [96].

In our study, MAT is a weak indicator of plant species richness (except for ferns) in simple liner regression models (Table 2). However, elevational patterns of MAT and species richness are different (the former had a monotonically decreased pattern whereas the latter had hump-shaped patterns) (Figures 3 and 4), indicating that assuming a linear relationship between MAT and species richness is not the most biologically reasonable hypothesis. On the contrary, a quadratic polynomial model may be more suitable for the relationship between MAT and species richness (Table 3). Furthermore, both MAT and MAP were highly correlated with species of all plant groups in final stepwise multiple regression (without MDE effect) (Table 2).

In summary, we suggest that the interaction influence of MAT and MAP may limit species richness at both extremes of the gradient, but in different ways, namely, at the lower end of the transect, temperature is high and precipitation is low; therefore high evapotranspiration and low humidity, consequently, limit the species growth, whereas at the upper end of the transect, species richness was limited by low temperature [31, 49]. We also concluded that there exists an optimum range of temperature and precipitation at the middle elevational area of Taibai Mountain and those favorable hydrothermal conditions may lead to higher energy available and therefore higher species richness [31, 49, 65]. Furthermore, McCain [92] proposed a climatic model and suggested that the optimal combination of temperature and water available may provide the most productive sites for more coexistence of species.

### 4.4. Rapoport's Elevational Rule

In this study, our results did not support Rapoport's elevational rule [11] in Taibai Mountain, whereas we supported the second hypotheses that range amplitudes are the highest in the middle of gradients [46]. One explanation for these observed patterns may be the random placement of species elevational ranges along an elevational gradient, like the MDE. Previous study also showed that the existence of MDE increased the difficulties to verify Rapoport's rule, and midpoint method may be influenced by the MDE [97]. In addition, different analysis methods used to test Rapoport's elevational rule were also a strong determinant of species richness patterns in mountain area [66, 68], as well as on latitude pattern [98]. Due to poor understanding of the complex factors determining range size [94], studies on the geographical patterns and decision-making mechanism of the species range still need to break through on the methods and means [93].

## 5. Conclusions

In Taibai Mountain, species richness of overall plants, seed plants, bryophytes, and ferns all demonstrated clearly hump-shaped patterns along the elevation gradient, although the absolute elevation of richness peaks varied somewhat. MAT and MAP were both main potential factors determining the richness patterns of each plant group in the regression models. In addition to climatic factors, MDE was also an important explanatory factor for bryophytes, while overall plants and seed plants were more related to area. Furthermore, Rapoport's elevational rule was not supported for any plant group. In this study, only spatial and climatic factors on elevational patterns of species richness were evaluated. However, we did not consider the historical evolutionary factors and human impacts on the elevational species richness patterns [51, 85, 99]. Further study on these factors might shed a light on the understanding of factors controlling elevational richness patterns of plant species.

## Figures and Tables

**Table 1:** Empirical species richness estimates for different elevational bands in Taibai Mountain.

Altitudinal band (m)	Bryophyte	Fern	Seed plants	Overall plants
800	20	24	14	58
900	28	29	80	137
1000	39	43	254	336
1100	52	42	319	413
1200	64	54	766	884
1300	81	61	767	909
1400	92	66	692	850
1500	111	73	656	840
1600	112	61	637	810
1700	122	61	611	794
1800	138	57	607	802
1900	139	51	593	783
2000	141	52	625	818
2100	132	43	501	676
2200	134	40	510	684
2300	132	40	504	676
2400	114	36	421	571
2500	120	38	415	573
2600	112	28	337	477
2700	115	29	304	448
2800	99	28	316	443
2900	93	22	222	337
3000	94	21	236	351
3100	91	9	178	278
3200	70	8	170	248
3300	56	4	127	187
3400	44	4	120	168
3500	40	4	145	189
3600	29	1	84	114
3700	1	1	64	66
All bands pooled	257	110	1491	1858

**Table 2:** Coefficients of determination (*R* ^2^) between species richness and explanatory variables for each plant group in simple linear regression analysis; standard coefficients (beta) for each variable and model fit (*R* ^2^) in stepwise multiple regression.

Plant groups	Variable	*R* ^2^ individual	Beta model A	Beta model B
Overall plants	Ln(area)	0.54∗∗∗	(*·*)	(*·*)
	MDE	0.45∗∗∗	−0.81∗	—
	MAT	0.24∗∗	(*·*)	0.26∗
	MAP	0.65∗∗∗	1.57∗∗∗	0.72∗∗∗
	Model fit (*R* ^2^)		0.72∗∗∗	0.71∗∗∗

Seed plants	Ln(area)	0.46∗∗∗	(*·*)	(*·*)
	MDE	0.36∗∗∗	−0.94∗	—
	MAT	0.26∗∗	(*·*)	0.30∗
	MAP	0.55∗∗∗	1.63∗∗∗	0.65∗∗∗
	Model fit (*R* ^2^)		0.65∗∗∗	0.63∗∗∗

Bryophyte	Ln(area)	0.82∗∗∗	(*·*)	(*·*)
	MDE	0.95∗∗∗	0.98∗∗∗	—
	MAT	0.01	0.11∗∗	−0.22∗∗∗
	MAP	0.92∗∗∗	(*·*)	1.03∗∗∗
	Model fit (*R* ^2^)		0.96∗∗∗	0.96∗∗∗

Fern	Ln(area)	0.37∗∗∗	(*·*)	(*·*)
	MDE	0.22∗	(*·*)	—
	MAT	0.60∗∗∗	0.61∗∗∗	0.61∗∗∗
	MAP	0.49∗∗∗	0.50∗∗∗	0.50∗∗∗
	Model fit (*R* ^2^)		0.82∗∗∗	0.82∗∗∗

Model A includes all variables; model B includes all variables except the MDE. (*·*) = variable excluded from analysis (*F*-significance > 0.1); — = variable not incorporated in model; ∗*P* < 0.05, ∗∗*P* < 0.01, and ∗∗∗*P* < 0.001.

**Table 3:** Series of second-order polynomial regressions for species richness and predictor variables.

Plant groups	Variable	Order	df	*R* ^2^	*b*_0_	*b*_1_	*b*_2_
Overall plants	Ln(area)	2	27	0.63∗∗∗	478.98	−219.83	27.15
	MDE	2	27	0.49∗∗∗	12.55	1.74	−0.001
	MAT	2	27	0.71∗∗∗	−210.59	244.83	−15.94
	MAP	2	27	0.65∗∗∗	729.66	−5.79	0.007

Seed plants	Ln(area)	2	27	0.53∗∗∗	374.60	−170.74	20.889
	MDE	2	27	0.41∗∗	−2.71	1.95	−0.002
	MAT	2	27	0.63∗∗∗	−173.81	185.07	−11.78
	MAP	2	27	0.55∗∗∗	113.53	−3.31	0.004

Bryophyte	Ln(area)	2	27	0.95∗∗∗	77.27	−37.26	4.71
	MDE	2	27	0.95∗∗∗	9.038	0.85	0.001
	MAT	2	27	0.96∗∗∗	−17.40	44.31	−3.31
	MAP	2	27	0.94∗∗∗	1274.03	−3.88	0.003

Fern	Ln(area)	2	27	0.42∗∗	27.11	−11.84	1.56
	MDE	2	27	0.24∗	9.24	1.37	−0.014
	MAT	2	27	0.82∗∗∗	−19.38	15.46	−0.84
	MAP	2	27	0.49∗∗∗	−657.91	1.39	−0.0007

Equation for second-order polynomials following equation term *f* = *b*_0_ + *b*_1_∗ variable + *b*_2_∗ variable^2^; df = degrees of freedom; ∗*P* < 0.05, ∗∗*P* < 0.01, and ∗∗∗*P* < 0.001.
